# Harnessing 3D-CT Simulation and Planning for Enhanced Precision Surgery: A Review of Applications and Advancements in Lung Cancer Treatment

**DOI:** 10.3390/cancers15225400

**Published:** 2023-11-14

**Authors:** Kazutoshi Hamanaka, Kentaro Miura, Takashi Eguchi, Kimihiro Shimizu

**Affiliations:** Division of General Thoracic Surgery, Department of Surgery, Shinshu University School of Medicine, Matsumoto 390-8621, Japan

**Keywords:** three-dimensional, computed tomography, lung cancer, precision surgery, simulation, navigation

## Abstract

**Simple Summary:**

Due to the increasing incidence of early-stage lung cancer and the demand for less invasive surgeries, sublobar resections are rapidly increasing in the field of lung cancer surgery. Recently, three-dimensional imaging technology and software solutions that enable highly precise lung segmentectomy simulations have been developed. In this article, we review reports on recent three-dimensional technology and its application in lung cancer surgery, introduce precision lung segmentectomies performed at our facility, and discuss its limitations. In the future, more accurate recognition of anatomical structures between the pulmonary artery, vein, and bronchus using artificial intelligence technology, visualization of anatomical structures using immersive technology such as extended reality, and devices that can be used intraoperatively without obstructing the operative field will be developed. If these advancements are realized, we believe that further high-quality tailor-made sublobar resections for lung cancer will be possible.

**Abstract:**

The clinical application of three-dimensional computed tomography (3D-CT) technology has rapidly expanded in the last decade and has been applied to lung cancer surgery. Two consecutive reports of large-scale prospective clinical trials from Japan and the United States have brought a paradigm shift in lung cancer surgery and may have led to a rapid increase in sublobar lung resections. Sublobar resection, especially segmentectomy, requires a more precise understanding of the anatomy than lobectomy, and preoperative 3D simulation and intraoperative navigation support it. The latest 3D simulation software packages are user-friendly. Therefore, in this narrative review, we focus on recent attempts to apply 3D imaging technologies, particularly in the sublobar resection of the lung, and review respective research and outcomes. Improvements in CT accuracy and the use of 3D technology have advanced lung segmental anatomy. Clinical applications have enabled the safe execution of complex sublobar resection through a minimally invasive approach, such as video-assisted thoracoscopic surgery and robotic surgery. However, currently, many facilities still render 3D images on two-dimensional monitors for usage. In the future, it will be challenging to further spread and advance intraoperative navigation through the application of 3D output technologies such as extended reality.

## 1. Introduction

The one-stage pneumonectomy performed by Graham in 1933 resulted in the first long-term survival after surgical treatment for lung cancer [1], and lobectomy was developed as a common procedure for lung cancer in the 1960s [2]. Since the inferiority of limited resection was reported by the Lung Cancer Study Group in 1995 [3], lobectomy has been the gold standard procedure for stage I non-small cell lung cancer (NSCLC) for several decades. However, the demand for sublobar resection for NSCLC has increased in recent years owing to the increase in the detection of small-sized lung cancer by screening computed tomography (CT) and research predicting noninvasive lung cancer from CT findings as the consolidation/tumor ratio [4].

The JCOG 0802/WJOG 4607L Japanese large-scale randomized trial, reported in 2022, prospectively enrolled patients with clinical stage IA NSCLC (tumor diameter ≤ 2 cm; consolidation-to-tumor ratio > 0.5). The proportion of local relapse was 10.5% in the segmentectomy group and 5.4% in the lobectomy group. However, overall survival (OS) was significantly better in the segmentectomy group (5-year OS rate: 94.3% in segmentectomy, 91.1% in lobectomy, hazard ratio 0.663) after a 7.3-year median follow-up time [5]. Furthermore, the results of the CALGB/Alliance 140503 trial conducted in the United States were reported the following year in the JCOG0802 report. In this trial, clinically staged as T1aN0 (tumor size ≤ 2 cm) NSCLC patients were randomly assigned to either sublobar resection (340 patients) including 201 wedge resection or lobar resection (357 patients). There was no statistically significant difference in 5-year disease-free survival (DFS) and OS after a 7-year median follow-up time, and the author concluded that sublobar resection was not inferior to lobectomy with respect to DFS and OS [6]. The results of both JCOG 0802/WJOG 4607L and CALGB/Alliance 140503 demonstrated the potential of sublobar resection for early-stage NSCLC.

The surgical anatomy of the lung, especially its segmental anatomy, has progressed in recent years owing to the development of high-resolution CT and three-dimensional (3D) technologies. This narrative review aimed to address the recent progress in the application of 3D technology to sublobar resection in lung cancer surgery and discuss its limitations and future directions.

We searched PubMed (https://pubmed.ncbi.nlm.nih.gov/ accessed on 12 April 2023) using the keywords “lung cancer”, “three-dimensional”, “segmentectomy” and “sublobar” for articles published between January 2010 and April 2023 to identify 3D-CT simulation for precise sublobar resection for lung cancer. Case reports, review articles, and editorials were excluded from the analysis. Furthermore, we added or excluded several studies as necessary for descriptive presentation in this review article.

## 2. Segmental Anatomy Analysis Using 3D-CT Images for the Lung

In the early studies of lung anatomy, the main method of investigation was autopsy, and inevitably the number of cases analyzed was relatively small. Recent anatomical studies of the pulmonary artery, vein, and bronchus, especially of the pulmonary segmental component, have been performed by converting high-resolution multidetector CT into 3D images, which has the advantage of being able to analyze a large number of cases with less invasiveness and cost, and the possibility of obtaining raw data from a living body inflated lung CT. The anatomy of the autopsy state and the living, breathing-synchronized state may be quite different, and an anatomical analysis using a breathing human body more accurately represents the pulmonary anatomical features.

Recent advances in techniques of pulmonary segmentectomy based on the studies of pulmonary segmental anatomy have been greatly influenced by the results using 3D-CT-based segmental anatomy studies conducted by Shimizu and his colleagues. Nagashima and Shimizu et al. used 3D-CT angiography and bronchography to analyze a bronchovascular pattern in a large number of cases and reported on the segmental anatomical pattern of the right upper lobe analyzed by 3D-CT of 260 [7] and 338 cases [8] consecutively. Preoperative 3D-CT simulation and intraoperative identification of the intersegmental veins are important in performing pulmonary segmentectomy, and their organization and patterning of the anatomy of the pulmonary vein have contributed greatly to the safe and high-quality procedures of segmentectomy. They particularly focused on the branching pattern of the central vein and classified the branching pattern of the pulmonary veins of the right upper lobe into four types: 1. Anterior with Central Iab type, 2. Anterior with Central Ib type, 3. Central type, and 4. Anterior type. The frequency of each type was 54, 26, 7, and 12%, respectively, and this classification is crucial because it may change the surgical strategy when performing a procedure of pulmonary segmentectomy. Moreover, Nagashima and Shimizu et al. reported anatomical patterns of the right middle and lower lobes as well as using 3D-CT in 270 patients [9].

Furthermore, Isaka et al. analyzed the segmental anatomy of the left upper lobe, focusing on the branching patterns of the bronchus and pulmonary veins using 3D-CT in 103 cases [10]. Deng et al. also analyzed the complex anatomical variations of the pulmonary artery, vein, and bronchus in a segment of the left upper lobe using 3D-CT in 103 cases and reported that preoperative 3D-CT reconstruction can help understand the anatomical variations and is effective for planning surgical procedures [11].

From a different perspective, Chan et al. reported a pilot study of newly developed software that permits automated segmentation of the pulmonary parenchyma, and although auto-segmentation was achieved in 72.7% of cases (32/44), the reasons for segmentation failure included local severe emphysema and pneumonitis [12]. Xu et al. proposed the concept of “lung surface intersegmental landmarks” based on an analysis of the distances between landmarks on the lung surface. They reported that although there were individual variations when stratified by sex, age, height, and weight, the proportions between these lengths remained constant, suggesting that this concept could be useful in determining the intersegmental planes for anatomic segmentectomy [13]. Furthermore, Chen et al. conducted a large-scale analysis of 800 cases, investigated the regularity of each segmental volume in the right upper lobe, and reported that the mean volume ratio of the anterior segment (S3) was the highest and that of the posterior segment (S2) was the lowest in the right upper lobe [14].

Several studies on the anatomical validity of pulmonary vessels and bronchi obtained from 3D simulations have been reported, and it has been shown that images converted to 3D maintain high accuracy. Hagiwara et al. constructed 3D images of 124 cases using Synapse Vincent software (Fujifilm Medical Co., Tokyo, Japan) and found that 97.8% of 316 branches of the pulmonary artery were consistent with intraoperative findings, excluding seven undetected branches of sizes less than 2 mm [15]. Cui et al. analyzed 52 cases using the original 3D image software “Exoview” and reported that 95.7% (132/138) of segmental pulmonary arteries, excluding six branches of sizes less than 1.4 mm, were precisely identified on reconstructed 3D images [16]. Nakao et al. performed a 3D simulation using REVORAS (Ziosoft, Inc., Tokyo, Japan) in 20 cases and reported that the simulated images of the bronchovascular anatomy were completely consistent with the intraoperative findings in 18 cases (90%) [17]. REVORAS is a next-generation software for 3D simulation that enables the simulation of a lung segmentectomy based on precise segmental anatomy and is used at our institute. Details of the clinical application of REVORAS at the Shinshu University Hospital are described later. Furthermore, Chen et al. reported a unique study in which an artificial intelligence (AI)-based chest CT semantic segmentation algorithm for pulmonary vessels at lobular or segmental levels achieved comparable accuracy to junior thoracic surgery attendings [18].

## 3. 3D-CT Simulation and Navigation for Sublobar Lung Cancer Surgery

Advances in 3D-CT technology and anatomy have facilitated the application of individual 3D images obtained from preoperative CT scans in clinical practice. Below, we review articles on 3D-CT simulations for clinical case series, 3D prints, virtual reality (VR), and other technologies for sublobar resection of lung cancer.

### 3.1. 3D-CT Simulation for Clinical Case Series

Our colleagues, Eguchi et al., reported 14 cases of intraoperative 3D-CT navigation segmentectomy performed from 2010 to 2011 using an iPad contained within a sterile bag for comparing the preoperative 3D-CT anatomy and the intraoperative anatomical structures, achieving sufficient surgical margins with no major postoperative complications [19]. Furthermore, Shimizu et al. discussed the importance of using preoperative 3D-CT simulation for video-assisted thoracoscopic surgery (VATS) segmentectomy to gain a better understanding of an individual patient’s segmental anatomy and anomalies, based on the findings obtained from their previous segmental anatomy analysis. They described that they always placed the 3D-CT image next to the thoracoscopic monitor during surgery, which allowed confirmation of the actual anatomical correlations with 3D-CT images, and also facilitated discussion between surgeons when there was a possible misconception of the anatomy [20].

Oizumi et al. reported the results of VATS segmentectomy in many patients with lung nodules using 3D-CT simulation, demonstrating its feasibility and safety, high completion rate of thoracoscopic procedures, sufficient surgical margins, and low recurrence rates [21,22,23,24]. She et al. compared the preoperative 3D simulation and “3D-VATS” using a 3D thoracoscopic system with 3D polarizing glasses during VATS segmentectomy with a two-dimensional (2D) group. They found that the operative duration, intraoperative bleeding, postoperative drainage, and air leakage were significantly reduced in the 3D group [25]. Xue et al. reported that 36 cases using 3D-CT simulation had a shorter operative time than 32 using conventional 2D-CT in VATS segmentectomy for lung cancer of less than 2 cm with ground-glass opacity (GGO), and they concluded that the guidance of 3D-CT simulation may enable accurately locate the GGO lesions and help with precise surgical planning compared with 2D-CT [26]. Wang et al. performed a large-scale study of 539 segmentectomies in a single institute using preoperative 3D-CT bronchography and angiography simulations, and modified inflation-deflation methods to identify intersegmental borders were used as effective alternatives to traditional methods [27]. A prospective multicenter randomized controlled trial (DRIVATS) involving a high-volume medical center in China was conducted; the primary endpoint in this trial was operation time, and the secondary endpoints included intraoperative blood loss, postoperative complications, and duration of hospitalization [28].

Preoperative 3D-CT simulation is beneficial even in complex segmentectomy, which was previously considered a highly difficult procedure. In general, segmentectomies are classified into two main categories: simple segmentectomy and complex segmentectomy. Simple segmentectomy is frequently performed because it is technically simple, such as segmentectomy for the left upper division, lingula, superior segment (S6), and basal segment. Other segmentectomies are classified as complex segmentectomies. Ohtaki et al. analyzed a case series of 53 cases of complex segmentectomy compared to 65 cases of simple segmentectomy for the left upper division and reported that complex segmentectomy resulted in less blood loss volume, less major complication, and similar surgical time and duration of postoperative chest drainage and concluded that complex segmentectomy can be performed safely under the guidance of 3D-CT simulation [29]. Zhang et al. assessed a case series including 42 cases of VATS anatomical complex segmentectomy for the basal segment and reported that 3D-CT simulation contributed to safe and feasible surgical procedures without conversion to open thoracotomy or lobectomy [30].

In the field of thoracic surgery, the surgical approach to lung cancer has evolved from the traditional open thoracotomy to a minimally invasive approach using VATS through a small 3- or 4-port incision that does not damage the thoracic muscle. In recent years, reduced port surgeries, such as uniportal VATS (u-VATS) with only one incision, have also been performed. Surgical robots initially developed for remote surgery are now available, and robot-assisted thoracic surgery (RATS) lung cancer surgery, as typified by the daVinci surgical system (Intuitive Surgical Inc., Sunnyvale, CA, USA), is becoming popular worldwide. Under such circumstances, 3D-CT simulations in lung segmentectomy have been applied to surgical procedures involving u-VATS and RATS. Le Moel et al. conducted a pilot study on RATS segmentectomy using the online 3D software, Visible Patient (Strasbourg, France), in nine cases between 2014 and 2015. They reported that 3D-CT reconstructed images of the chest wall and ribs were also useful for simulating optimal port placement and the relationship between the tumor and the chest wall [31]. Zheng et al. investigated 71 cases of u-VATS segmentectomy utilizing intraoperative intersegmental plane management by trimming the 3D pulmonary structure along the intersegmental demarcation using an ultrasonic scalpel based on 3D-CT simulation, the “Combined Dimensional Reduction method” (CDR method) and reported that the operation time and postoperative hospital stay were shortened compared to those in conventional procedures. Moreover, they analyzed the recovery rate of postoperative forced expiratory volume in 1 s (FEV1) at 1 and 3 months after segmentectomy and found that the CDR group was slightly better than the non-CDR group, although not statistically significantly different [32]. Matsuura et al. compared uniportal VATS and multiportal VATS for common and uncommon segmentectomies with preoperative 3D-CT simulations and reported that 45 cases of u-VATS (including 22 cases of uncommon segmentectomy) had a shortened operation time and postoperative hospitalization [33].

One of the important advantages of segmentectomy utilizing 3D-CT simulation is the achievement of a free surgical margin. Securing the resection margin is essential for the surgical resection of malignant tumors, and the margin distance required depends on the type and characteristics of the tumor, such as primary lung cancer or metastatic lung tumor, hypermetabolic solid type tumor, or GGO-predominant type hypometabolic tumor. When the tumor is located in the periphery of the lung, it is important to secure the margin during the dissection of the lung parenchyma, whereas when the tumor is located in the central part of the lung, preoperative simulation and intraoperative navigation of the pulmonary artery, vein, and bronchial structures utilizing 3D-CT are important. Therefore, we reviewed several studies describing the surgical margin in the context of 3D-CT simulation. Nakamoto et al. performed 30 cases of segmentectomy using 3D-CT for lung nodules, including metastatic or benign tumors located deeper than 20 mm from the pleural surface, and all the cases had secured surgical margins [34]. Kanzaki et al. performed multisubsegmentectomies, such as right S^2^b + S^3^a, left S^1+2^a + b, or others, using VATS in 11 patients using a virtual 3D pulmonary model with CTTRY, a custom software they developed, and reported that all the patients had negative surgical margins [35]. Wang et al. compared 92 cases of VATS complex segmentectomy divided into 42 cases in the 3D group and 55 in the routine group and reported that all the cases in the 3D group, in which preoperative 3D-CT reconstruction was performed, had adequate surgical margin of >2 cm [36]. Iwano et al. defined the virtual safety margin as a sphere extending 2 cm outside the tumor in preoperative 3D-CT simulation and reported that no positive surgical margin was achieved in all 17 primary lung cancers in 16 patients after segmentectomy [37]. Wu et al. similarly reported 57 cases of VATS segmentectomy with preoperative 3D-CT simulation with a virtual 3D surgical margin defined as a sphere extending at least 2 cm outside the lesion or 2 cm greater than the tumor size and concluded that the combination of VATS and 3D-CT provided a transition from lesion-directed location of tumors to computer-aided surgery for the management of early lung cancer [38]. Xu et al. reported that 61 cases of u-VATS segmentectomy with intersegmental plane simulation by 3D reconstruction achieved a gross margin sufficiency of 100%, whereas 5 of 59 cases without intersegmental plane simulation did not achieve margin sufficiency [39].

The use of preoperative 3D simulation led to better outcomes in many matters, including less intraoperative bleeding and chest tube duration [25], shorter operation time [26], fewer stapler reloads, and the reduction of postoperative air leakage on postoperative days 1–3 [36] compared to the cases without 3D simulation.

### 3.2. 3D Output Technologies from 3D-CT Reconstruction Images

In many previous clinical studies and case reports, reconstructed 3D-CT simulation images were displayed on a 2D monitor. However, displaying the image data of 3D-CT images in a 3D format may enhance the intuitive anatomical understanding.

Liu et al. reported that the 3D group (3D printing and 3D-CT) showed better surgical outcomes (intraoperative blood loss, operation time, and postoperative hospital stay) than the general (2D) group; however, there was no significant difference between the 3D-print and 3D-CT groups for experienced surgeons [40]. Qui et al. conducted a similar comparison between 3D reconstruction, a 3D printed model, and non-3D groups and reported that while there were no differences in outcomes among the three groups in simple segmentectomy, the 3D-printed model group showed shorter operation time and lower intraoperative blood loss in complex segmentectomy. Additionally, using a questionnaire survey, they reported that the surgeons were satisfied with the clinical effectiveness of the 3D-printed model [41]. Furthermore, several studies have reported improved surgical outcomes (such as reduced operation time, blood loss, and conversion to lobectomy) in segmentectomy by utilizing 3D-printed models rather than 3D-CT reconstruction images on 2D monitors [42,43,44]. By adding a 3D-print model to the conventional 3D-CT simulation, the rates of approach transfer and conversion to lobectomy [42], intraoperative blood loss [42,43], and operation time [43,44] were reduced. Furthermore, 3D-print simulations reduced operation time and intraoperative blood loss, even in complex segmentectomies [41].

Another format for outputting 3D simulations is 3D stereoscopic vision using glasses-type devices such as polarized glasses and VR headsets. Kanzaki et al. reported 10 cases of 3D thoracoscopic sublobar resection (3D TSLR), in which they used a 3D endoscopic monitor while wearing 3D polarized glasses and simultaneously displaying 3D simulation images on a stereoscopic vision display for intraoperative navigation [45]. Ujiie et al. reported a case of a left upper segmentectomy utilizing a VR surgical navigation system that involved loading 3D polygon data into the Banana Vision software (Colorado State University) and displaying it on head-mounted displays (HMD) [46].

Studies on the additional use of 3D-CT for intraoperative navigation were reported by Chang SS. Chang et al. performed 12 cases of VATS segmentectomy by clipping titanium clips on the surface of the intersegmental plane visualized by the ventilation and collapse method and obtained 3D-CT with a C-arm CT in a hybrid operating room to confirm the correct anatomical segmental border [47]. Furthermore, Chang et al. subsequently reported that 34 cases of VATS segmentectomy from a different institution achieved complete resection by 3D-CT navigation using intraoperative cone-beam CT combined with the indocyanine green (ICG) fluorescence method and intersegmental line marking by titanium clipping [48].

### 3.3. 3D Simulation-Based Precision Sublobar Resection at Shinshu University

**Figure 1:**
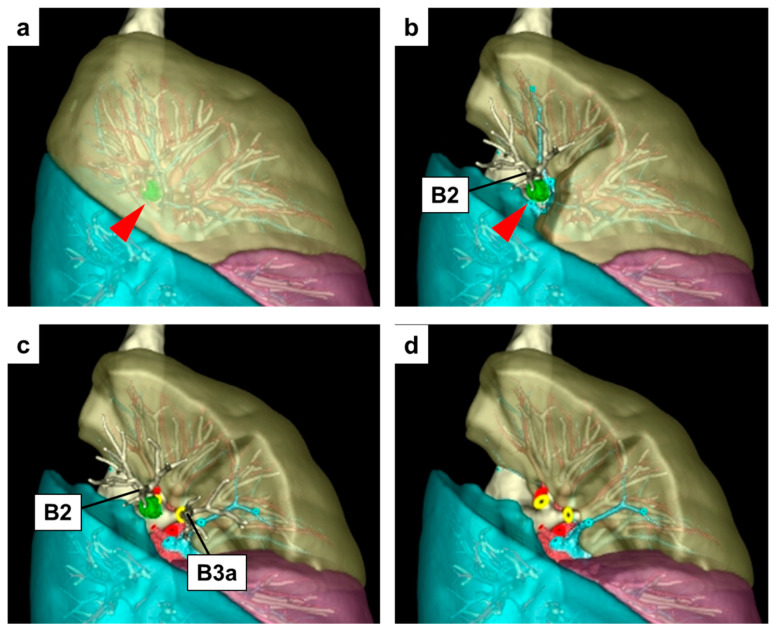
Preoperative 3D-CT simulation for right S2+3a subsegmentectomy using REVORAS. 3D-CT image of right upper lobe (**a**). The lung tumor is shown in green with a red arrowhead. The simulation image of the lung parenchyma after S2 segmentectomy for the right upper lobe without sufficient surgical margin (**b**). The B3a dissection was added, and the S2 + 3a subsegmentectomy was simulated to secure a sufficient surgical margin (**c**). The divided vein (blue circle), artery (red circle), and bronchus (yellow circle) are shown after the right S2+3a subsegmentectomy (**d**).

In our department at Shinshu University, we used the latest 3D software, REVORAS, developed by Zaiosoft, to create 3D images of all the patients scheduled for anatomical lung resection. For patients undergoing sublobar resection, we performed a preoperative simulation according to the individual segmental anatomy, tumor location, features of pulmonary nodules, and adequate surgical margins. With REVORAS, it is possible to simulate the dissection of the bronchus or pulmonary artery at arbitrary points, enabling complex simulations regardless of the unit of lobe or segment of the lung. We used preoperative simulation for intraoperative navigation [49]. We present the actual preoperative 3D-CT simulation images using REVORAS of surgical cases performed at our department. In this case, the tumor was located on the right S2 and close to S3, and only the S2 segmentectomy could be inappropriate, so a simulation of S2 + 3a resection was performed to obtain a sufficient surgical margin distance (Figure 1a–d).

**Figure 2:**
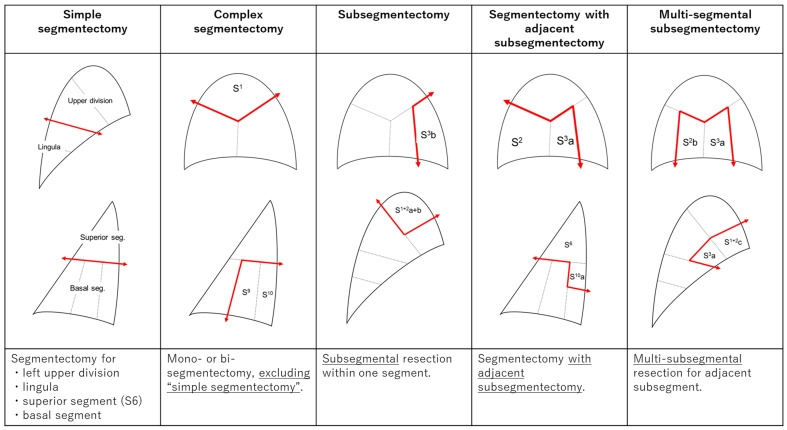
Sublobar resection is classified into five categories. The types of the procedure are described at the top. The schemas of representative procedures are shown in the middle. The lower part shows the explanation of the contents of the procedure.

**Figure 3:**
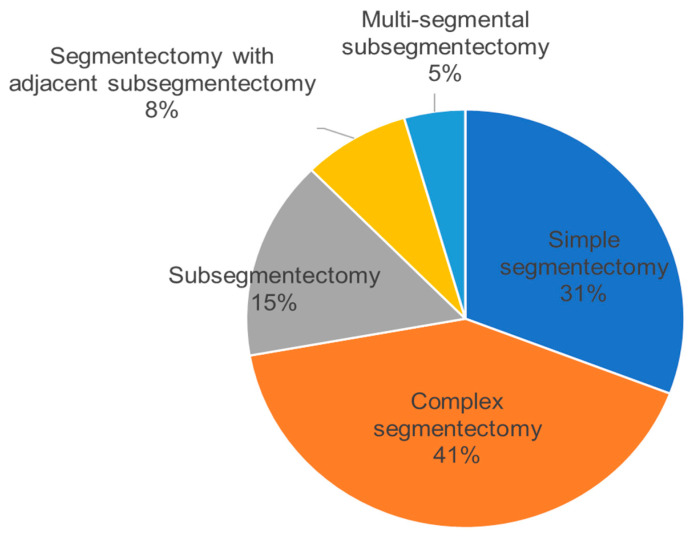
Pie chart: shaded sectors correspond to the percentages of each type of procedure defined in Figure 1.

We classified sublobar resection into five categories, excluding wedge resection performed in our department, and have provided the corresponding schema and explanations in Figure 2. We present detailed data on sublobar resections performed in our department between August 2019 and September 2022 (Table 1) and the distribution of these cases (Figure 3). We thus performed not only “simple segmentectomy” and ordinary “complex segmentectomy”, but also “subsegmentectomy” for reducing resection volume if sufficient margin distance can be secured, taking into consideration the tumor location, tumor size, and the component of GGO and solid part as the consolidation-to-tumor ratio (CTR). In cases where the tumor is close to the intersegmental border and the resection margin may be inadequate, we perform “segmentectomy with adjacent subsegmentectomy” to secure the sufficient surgical margin distance. Moreover, if the surgical margin distance is maintained for small tumors located on the segmental border, a “multi-segmental subsegmentectomy” is performed by combining subsegmental resection of the adjacent segment. Under this policy for pulmonary segmentectomy for lung malignancy, the mean number of resected segments in the 266 cases shown in Table 1 was 1.34, which is a rather small number of segments (if the subsegment is calculated uniformly as 0.5 of a segment). We conducted tailor-made complex sublobar resections specifically designed to suit each patient.

To deliver the highest level of personalized care for each patient, we have instituted a four-pronged approach known as the 3D-S strategy. The 3D-S strategy encompasses four key steps: Developing, Demonstrating, Discussing, and Sharing. “Developing” involves crafting bespoke 3D-CT-based surgical plans for each patient. These plans are not just a visualization tool but a roadmap for the surgical team, detailing every case’s unique anatomical and surgical considerations. “Demonstrating” involves presenting these 3D-CT-based plans to the surgical team through interactive sessions. This allows the team to visualize the complexities of each case, enabling the team to anticipate and prepare for specific challenges. In the “Discussing” phase, the surgical team comes together to discuss the plan collaboratively. This is a crucial step for preempting potential complications and preparing for any contingencies during surgery. The final step, “Sharing”, ensures that the refined 3D-CT plans are disseminated among all team members. This guarantees that everyone, from the lead surgeon to the assisting medical staff, is aligned in understanding the surgical strategy. Additionally, we offer specialized simulation training programs tailored to each patient’s planned procedure. These programs serve as a rehearsal for the surgical team, ensuring they are fully prepared and confident in executing the plan. This comprehensive 3D-S approach is integral to our commitment to providing the best possible outcomes for our patients [50].

## 4. Conclusions and Future Directions

Recent technological innovations have been introduced into the medical field, with advances in CT equipment and 3D reconstruction, and have further evolved into technologies such as 3D printing and VR, enabling visual outputs in 3D. The development of the 3D segmental anatomy of the lung and its application to surgical simulation has facilitated complex and precise sublobar resection, enabling minimal resection of malignant tumors such as lung cancer and metastatic lung tumors. Many studies have reported that sublobar resection using 3D technology leads to better outcomes than surgery without 3D simulation.

The limitation of 3D simulation is the necessity of introducing expensive 3D simulation software packages. Moreover, the accuracy of current CT equipment for detecting extremely small pulmonary vessels is limited. Furthermore, in many facilities, images reconstructed in 3D are displayed on 2D monitors for preoperative use. However, to enhance the effective utilization of 3D-CT for simulations and navigation, it is desirable to output stereoscopic 3D images. Although 3D printing is a viable method, it requires individual manufacturing and is unsuitable for storage. Outputting 3D data in VR may solve this issue; however, further development in technology such as extended reality (XR) that covers VR, augmented reality, mixed reality, or other immersive technologies is necessary for its utilization in intraoperative navigation, which does not interfere with surgical procedures, is naturally and unobtrusive, and could become the ultimate form of intraoperative 3D navigation.

This review focused on the paradigm shift in the field of lung cancer surgery, with a particular emphasis on the utilization of 3D-CT simulation in “precision” surgery with recent advances in lung-preserving sublobar resection. Results of recent clinical trials challenge the traditional approach of lobectomy for early-stage lung cancer and show that sublobar resection is equally effective in improving survival. Various topics related to 3D-CT lung segmental anatomy for precision surgery were also discussed, as well as the latest advances in simulation and navigation techniques to guide surgeons to successful resection for lung segments. Overall, this review will be useful to those interested in the latest research and advances in lung cancer surgery by highlighting exciting developments in the field of 3D-CT simulation and the potential for 3D-CT navigation for precise sublobar resection to improve patient outcomes.

## Figures and Tables

**Table 1:** Detailed data on sublobar resections performed at Shinshu University. The surgical procedure and the number of cases are shown. The bottom row shows the total number and proportion of each type of procedure.

Simple Segmentectomy		Complex Segmentectomy		Subsegmentectomy		Segmentectomy with Adjacent Subsegmentectomy		Multi-Segmental Subsegmentectomy	
left upper devision	15	S^1^	12	S^1^a	4	S^1+2^ + S^3^a	2	S^1^a + S^2^a	1
Lingula	15	S^1^+ S^2^	2	S^1+2^b	2	S^1+2^ + S^3^a+c	1	S^2^b + S^3^a	4
Lingula + S^6^	1	S^1^ + S^4^	1	S^1+2^c	2	S^2^ + S^1^a	1	S^1+2^a+b + S^3^c	1
Lingula + basal seg.	1	S^1^ + S^9-10^	1	S^1+2^a+b	3	S^2^ + S^3^a	3	S^1+2^c + S^3^a	1
S^6^	37	S^1+2^	11	S^1+2^a+c	1	S^3^ + S^1+2^a	2	S^1+2^c + S^3^c	1
Basal seg.	13	S^1+2^ + S^6^	1	S^1+2^b+c	1	S^3^ + S^4^+S^5^a	1	S^1+2^c + S^3^a + S^4^a	1
		S^2^	11	S^2^b	1	S^4^ + S^3^b	3	S^3^a + S^4^a	1
		S^3^	20	S^3^b	10	S^4^ + S^3^a+b	1	S^8^a+S^9^a	1
		S^3^ + S^4+5^	1	S^3^b+c	1	S^4^+S^5^+S^1+2^c	1	S^8^a+S^9^a + S^3^b+c	1
		S^4^	4	S^4^a	2	S^6^ + S^10^a	2		
		S^5^	5	S^5^a	1	S^6^ + S^8^a+S^9^a	1		
		S^5^ + S^8^	1	S^5^b	1	S^8^ + S^9^b	1		
		S^6^ + S^10^	1	S^6^a	1	S^10^ + S^6^c	1		
		S^7^	6	S^6^b	1	S^10^ + S^7^b	1		
		S^7^+S^8^	1	S^8^b	5				
		S^7^+S^10^	1	S^9^b	1				
		S^8^	5	S^10^b+c	4				
		S^8^+S^9^	6						
		S^8^+S^9^+S^10^	3						
		S^9^	5						
		S^9^+S^10^	7						
		S^10^	5						
	82 (31%)		110 (41%)		41 (15%)		21 (8%)		12 (5%)
